# The disappearance of IPO in myocardium of diabetes mellitus rats is associated with the increase of succinate dehydrogenase-flavin protein

**DOI:** 10.1186/s12872-021-01949-z

**Published:** 2021-03-17

**Authors:** Mengyuan Deng, Wei Chen, Haiying Wang, Yan Wang, Wenjing Zhou, Tian Yu

**Affiliations:** 1 Department of Anesthesiology, Affiliated Hospital of Zunyi Medical University, 149 Dalian Road, Huichuan District, Zunyi, 563003 Guizhou People’s Republic of China; 2 Anesthesia Laboratory, Affiliated Hospital of Zunyi Medical University, Zunyi, People’s Republic of China; 3 Zunyi Medical University, Zunyi, People’s Republic of China

## Abstract

**Background:**

The aim of the present study was to investigate whether the disappearance of ischemic post-processing (IPO) in the myocardium of diabetes mellitus (DM) is associated with the increase of succinate dehydrogenase-flavin protein (SDHA).

**Methods:**

A total of 50 Sprague Dawley rats, weighing 300–400 g, were divided into 5 groups according to the random number table method, each with 10 rats. After DM rats were fed a high-fat and -sugar diet for 4 weeks, they were injected with Streptozotocin to establish the diabetic rat model. Normal rats were fed the same regular diet for the same number of weeks. Next, the above rats were taken to establish a cardiopulmonary bypass (CPB) model. Intraperitoneal glucose tolerance test (IPGTT) and oral glucose tolerance test (OGTT) were used to detect whether the DM rat model was established successfully. Taking blood from the femoral artery to collect the blood-gas analysis indicators, and judged whether the CPB model is established. After perfusion was performed according to the experimental strategy, the area of myocardial infarction (MI), and serum creatine kinase isoenzyme (CK-MB) and cardiac troponin (CTnI) levels were measured. Finally, the relative mRNA and protein expression of SDHA was detected.

**Results:**

The OGTT and IPGTT suggested that the DM rat model was successfully established. The arterial blood gas analysis indicated that the CPB model was successfully established. As compared with the N group, the heart function of the IR group was significantly reduced, the levels of myocardial enzyme markers, the area of MI, as well as the relative mRNA and protein expression of SDHA, were all increased. As compared with the IR group, the CK-MB and CTnI levels in the IPO group, the MI area, relative mRNA and protein expression of SDHA decreased. As compared with the IPO group, the myocardial enzyme content in the DM + IPO group, the MI area and the relative mRNA and protein expression of SDHA increased. As compared with the DM + IPO group, in the DM + IPO + dme group, the myocardial enzyme content, area of MI and relative mRNA and protein expression were all decreased.

**Conclusion:**

IPO can inhibit the expression of SDHA, reduce MIRI and exert a cardioprotective effect in the normal rats. However, the protective effect of IPO disappears in the diabetic rats. The inhibitor dme combined with IPO can increase the expression of SDHA and restore the protective effect of IPO in DM myocardia.

**Supplementary Information:**

The online version contains supplementary material available at 10.1186/s12872-021-01949-z.

## Introduction

Myocardial ischemia–reperfusion injury (MIRI) is the pathological core of several cardiovascular diseases, such as stroke and myocardial infarction (MI) [1]. Its factors are multifaceted, such as the production of reactive oxygen species (ROS) during reperfusion [2], and the opening of the permeability transition pore [3] and activation of nuclear factor erythroid 2-related factor 2 [4]. Ischemia post-processing (IPO) can induce the miR-21 regulation axis through hypoxia-inducible factor 1-α, downregulate the inflammatory mediators programmed cell death protein 4 and Fas ligand, inhibit inflammatory response and exert myocardial protection [5]. In the diabetes mellitus (DM) myocardium, the anti-MIRI effect of IPO is weakened or disappears [6, 7]—which may be due to the decreased expression modulation of cystathionine γ-lyase [8]—phosphorylate extracellular signal related kinase fails [9], phosphoinositide-3-kinase (PI3K) is activated [10], the mechanistic target of rapamycin is upregulated [11] and autophagy is reduced. But the specific mechanism remains unclear. SDH, also known as succinate ubiquinone oxidoreductase or mitochondrial complex II, is the only multi-subunit enzyme integrated in the inner mitochondrial membrane of the tricarboxylic acid cycle (TCA). It is composed of two water-soluble heterodimers of dehydrogenase-flavin protein (SDHA) and succinate dehydrogenase-iron-sulfur protein (SDHB), combined with two hydrophobic subunits SDHC (CybL) and SDHD (Cybs). However, as the only active group in the SDH structure, SDHA is a key factor in coupling mitochondrial electron transport and respiratory function, while SDHB, C, D, etc. are inactive groups that only play a role in maintaining structural stability [12]. Therefore, this experiment focused on SDHA. When SDH is mutated, it causes massive accumulation of succinic and fumaric acid [13], adenine nucleotide decomposition [14], partial reversal of the malic acid/aspartic acid shuttle process and the opening of the hypoxic signal pathway. When the mitochondrial self-storage breathing capacity disappears completely, a large amount of ROS is generated, resulting in myocardial cells death [15].

SDH competitive inhibitor-dimethyl malonate (dme) can reduce the accumulation of succinic acid and reduce oxidation, and finally decrease MIRI [16–18]. Our previous study found that when MIRI occurs, the expression of SDHA increases and is two times higher than that of the control group, and that IPO can reduce the expression of SDHA and reduce MIRI [19]. Based on the results of this study, we speculated that SDHA overexpression may be involved in MIRI, and the cardioprotective effect of IPO may be associated with the inhibition of SDH. The increased oxidative damage response or energy metabolism disorder caused by the increased expression of SDHA may be the main reason for the reduced anti-MIRI effect of IPO in the DM myocardium, and dme may restore the myocardial protective effect of IPO in the DM myocardium.

## Materials and methods

### Animal experiment group

**Fig. 1:**
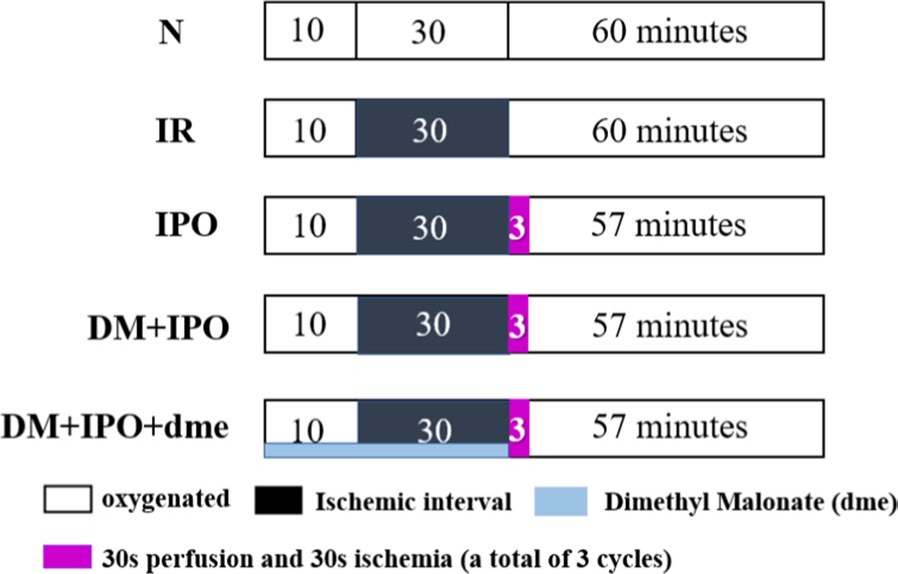
Grouping and perfusion strategy in rats. A total of 50 standard male Sprague–Dawley rats were randomly divided into 5 groups (n = 10, each group): The N, IR, IPO, DM + IPO and DM + IPO + dme groups. DM, diabetes mellitus; N, normal; IR, ischemia–reperfusion; *IPO* ischemia post-conditioning, *DM* + *IPO* DM ischemia post-conditioning, *DM* + *IPO* + *dme* DM ischemia post-conditioning with inhibitor

A total of 50 rats were divided into five groups according to the random number table method (ten rats in each group): The normal (N), ischemia reperfusion (IR), ischemic post-treatment (IPO), diabetic mellitus ischemia postconditioning (DM + IPO) and ischemic postconditioning of diabetic rats and combined of inhibitor group (dme is an inhibitor of SDHA), which is abbreviated as (DM + IPO + dme) groups (Fig. 1).

### Materials

Dimethyl malonate (dme), streptozocin, citric acid and sodium citrate were purchased from Merck KGaA. Rabbit SDHA antibody (cat. no. EPR9043) was purchased from Abcam. Rabbit GAPDH antibody (cat. no. EPR16891) was purchased from Abcam. HRP Goat anti-rabbit secondary antibody was purchased from Abcam (cat. no. ab205718). Reverse transcription kit SYBG-QPCR kit was purchased from Vazyme. The primers were purchased from Jiangsu Synthgene Biotechnology Co, Ltd.. Succinate acid, rotenone and adenosine diphosphate was purchased from Beijing Solarbio Science & Technology Co., Ltd..

### Establishment of DM rat model

Adult male Sprague Dawley (SD) rats (age, 16–20 weeks; weight, ~ 300 g) were purchased from Changsha Tianqin Biotechnology Co., Ltd. [license no., SCXK (Xiang) 2014-0011]. All experimental procedures involving animals were complied with the document No. 233 of the Animal Ethics Committee of Zunyi Medical University (approval no., ZMUER2016-2-233). No animals were specifically generated or sacrificed for this study. The experiment was carried out in compliance with the ARRIVE guidelines 2.0 (Nathalie Percie du Sert, Amrita Ahluwalia, Sabina Alam, July 14, 2020). DM rats were fed with a high-fat and -sugar diet for 4 weeks (basic diet 60%, lard 18%, white sugar 20% and egg yolk 2%). Ordinary rats were fed with ordinary feed for the same number of weeks (basic feed 60%, protein 33%, oil 3%, amino acid and mineral 4%). Four weeks later [20], according to the dose of 35 mg/kg, 1% STZ solution was injected into the tail vein of DM rats, and the blood glucose level of the tail vein was measured 72 h later. If it was more than 16.0 mmol/l, the DM rat model was considered to have been successfully established [14].

### Establishment of rat cardiopulmonary bypass (CPB) model

First, 2% pentobarbital solution (50 mg/kg) was injected into the intraperitoneal cavity. After the rats were anesthetized, they were fixed on the plates. A 16G trocar was then used for tracheal intubation by connecting it to a small animal ventilator and setting the breathing parameters as: Respiratory rate, 60 times/min; tidal volume, 3 ml/kg; breathing ratio, 1:1.5; mechanical ventilation. The rat model oxygenator, blood reservoir and peristaltic pump were connected, and the tubing was prefilled with prefilled fluid (including hydroxyethyl starch, sodium potassium magnesium calcium glucose injection and mannitol solution). The left femoral artery (a tube was inserted to monitor arterial blood pressure), right femoral artery (CPB blood return), and tail vein (rehydration and administration) were separated. The right jugular vein was separated and a tube was placed at the junction of the right atrium and the inferior vena cava (pre-experiments have confirmed that the drainage is the most easiest and smoothest here). After the tube had been placed, the skin was cut longitudinally along the sternum, the skin, was cut along the middle (parallel to the level of the third rib) to expose and aortic root. Heparin (4 mg/kg) was injected through the tail vein. When ACT was at > 480 s, the right jugular vein was opened for perfusion, and the target flow was adjusted to 100 ml/kg·min. At this time, the CPB model was considered successful. A mean arterial pressure (MAP) of > 60 mmHg was maintained during the experiment.

In the N group, perfusion was maintained at 100 ml/kg·min for 100 min and then the experiment was ended. In the IR group, the aforementioned flow perfusion was maintained for 10 min, the overhead light and insulation blanket were turned off, the aortic root was clamped so that the heart was arrested for 30 min, then the aorta was rewarmed and opened, heart re-beat was implemented, and the experiment was ended after 60 min of perfusion. In the IPO group, based on the bypass strategy of the IR group, at the end of reperfusion, 30 s ischemia-30 s perfusion was performed for a total of 3 cycles, the aorta was opened, and the experiment was ended after 57 min at the same flow as before. In the DM + IPO group, DM rats were used, and the diversion strategy was the same as that for the IPO group. In the DM + IPO + dme group, the SDH inhibitor dme (4 mg/kg·min) was continuously pumped from the tail vein 10 min before the aorta was blocked.

### Detection index

#### Oral glucose tolerance (OGTT) and intraperitoneal glucose tolerance (IPGTT) determination

Using DM and normal rats, the 20% GS solution was administered by gavage and abdominal cavity. During the gavage and abdominal cavity, tail vein blood was collected immediately, and 30, 60 and 90 min later, and the blood glucose value was recorded in each rat.

### Arterial blood gas analysis

Using the rats in the N group, arterial blood was collected 5 min before CPB (T0), at the beginning of CPB (T1), at cardiac arrest (T2), at the end of cardiac arrest (T3), after 5 min of reperfusion (T4) and at the end of reperfusion (T5), respectively. The results of blood gas analysis were then recorded, and it was judged whether the CPB model had been successfully established. The rats in the N group did not undergo cardiac arrest with relapse, so arterial blood only needed to be collected at the corresponding time point.

### Heart function data

Mean arterial pressure (MAP) and heart rate (HR) were recorded at 5 min before CPB (T0), the start of CPB (T1), cardiac arrest (T2), end of cardiac arrest (T3), 5 min of reperfusion (T4) and the end of reperfusion (T5).

### Detection of MI (myocardial infart) area

The detection were used five groups, each with four myocardial tissues. At the end of reperfusion (T5), the entire myocardial tissue was quickly removed, and the blood was washed with PBS solution, and placed in a refrigerator at − 80 °C for 10 min. A 1% 2, 3, 5 triphenyltetrazolium chloride (TTC) solution was prepared avoiding the light, and placed in a 37 °C water bath. The frozen myocardial tissue was collected, and the heart was quickly cut into 5 tissues of equal thickness from the apex to the bottom of the heart, which were placed in the TTC solution, incubated in the dark for 30 min, and fixed with 10% formaldehyde for 24 h. The slices were arranged from small to large, the slices were photographed and the MI area was calculated using ImageJ software; the percentage of white (myocardial infarct area) in the total area of the myocardium was used as the result for statistics.

### Detection of creatine kinase isoenzyme (CK-MB) and cardiac troponin (CTnI)

At T5, 1 ml blood was collected and left to stand at room temperature as the sample to be tested. The centrifuge was pre-cooled, and the parameters set to 3000×*g* for 20 min. The blood samples were centrifuged, the supernatant separated and the CK-MB and CTnI detection kit (Biosco) was used for detection, according to the ELISA method.

### Detection of the relative mRNA expression of SDHA by RT-qPCR

The step requires 5 sets of samples, each with 6 hearts in rats. At the end of reperfusion (T5), the left ventricular tissue of the rat was quickly removed, and the blood was washed and placed in a refrigerator at − 80 °C. First, the Trizol method was used to extract total RNA. Secondly, the Thermo Fisher multifunctional microplate reader (Thermo Fisher Scientific, Inc.) was used to measure the OD260 and OD280 of each sample. When the ratio was in the range of 1.8–2.1, the sample was considered to be of high purity and subsequent experiments could be performed. Then, the sample was tested and the Bio-rad thermal cycler was used to perform the reverse transcription reaction in the order of 50 °C for 15 min and 85 °C for 5 s; Following the reaction, the cDNA was placed in the − 80 °C refrigerator for later use. Primers were prepared for each target gene, Vazyme reverse transcription kit (Novizan Biotechnology Co., Ltd.) was used to configure the reaction system; it was placed on a PCR machine (Analytik Jena AG), and underwent pre-denaturation (95 °C for 30 s), cycle reaction (95 °C for 10 s; 60 °C for 30 s) and dissolution reaction (95 °C for 15 s; 60 °C for 60 s; 95 °C for 15 s). The relative expression of the target gene was calculated and analyzed according to the sample Cq value.

The primer sequence for each gene was as follows: SDHA, 5′-AGCCTCAAGTTCGGGAAAGG-3′ forward and 5′-CACAGTGCAATGACACCAAC-3′ reverse. GAPDH, 5′-AGTGCCAGCCTCGTCTCATA forward and 5′-GGTAACCAGGCGTCCGATAC-3′ reverse.

### Detection of the relative protein expression of SDHA by western blotting

The steps required 5 sets of samples, each with 6 hearts in rats. First, RIPA lysate (Solebold) and protease inhibitor PMSF (Solebold) were used to extract protein. Next, using SDS-PAGE gel, following loading, electrophoresis, membrane transfer, blocking, primary antibody incubation and membrane washing (SDHA primary antibody dilution concentration, 1:1000), secondary antibody incubation and membrane washing (GAPDH concentration, 1:3000), the ECL exposure solution was dropped evenly on the PVDF membrane and left to stand for 1 min. The strips were exposed with ChemiDoc gel imaging system (Bio-Rad Laboratories, Inc.), and the exposed images were finally analyzed with image analysis software.

### Data analysis

Experimental data was collected in accordance with the principle of complete, random and control, and recorded as the mean ± standard deviation (${\bar{X}}\pm{\text{S}}$). SPSS 17.0 was used for statistical analysis. A single-factor analysis of variance was used for intra-group comparisons, and comparisons within different time points were performed using repeated measures analysis of variance. The LSD and the S–N–K methods were used for uniform variance, and the Dunnett’s T3 method for uneven variance. P < 0.05 was considered to indicate a statistically significant difference.

## Results

### OGTT

**Fig. 2:**
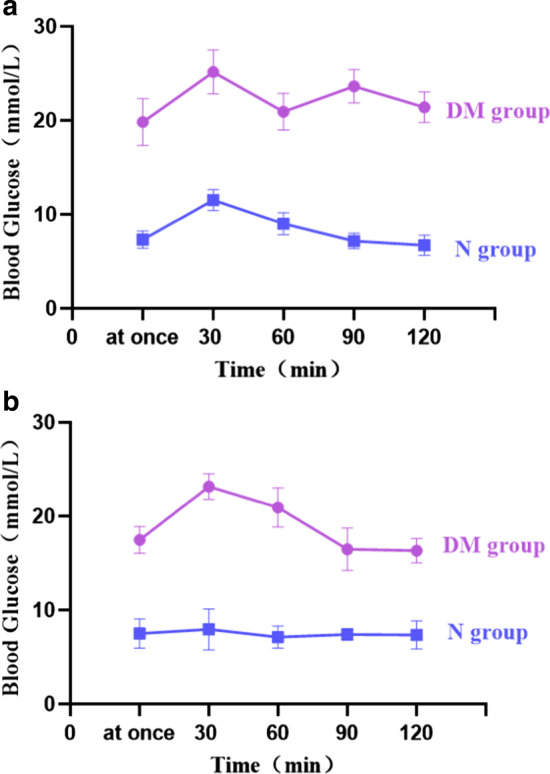
Comparison of IPGTT and OGTT in each group of rats. **a** OGTT. **b** IPGTT. All data are expressed as the mean + SD. *DM* diabetes mellitus, *N* normal, *IPGTT* intraperitoneal glucose tolerance test, *OGTT* oral glucose tolerance test

Both normal and DM rats had blood glucose peaks 30 min after gavage, and then slowly decreased. The blood glucose of ordinary rats was at 7 to 11 mmol/l during the whole process. As compared with ordinary rats, the blood sugar of DM rats was maintained at a higher level during the whole process (20–25 mmol/l; Fig. 2A).

### IPGTT

In normal rats, blood glucose levels peaked 30 min after intraperitoneal injection, but then slowly decreased and stayed at 7–8 mmol/l throughout the entire process. As compared with ordinary rats, the blood sugar of DM rats was in a state of hyperglycemia (> 16 mmol/l) throughout the whole process (Fig. 2B).

### Changes in cardiac function data

#### Changes in the MAP of rats in each group

**Fig. 3:**
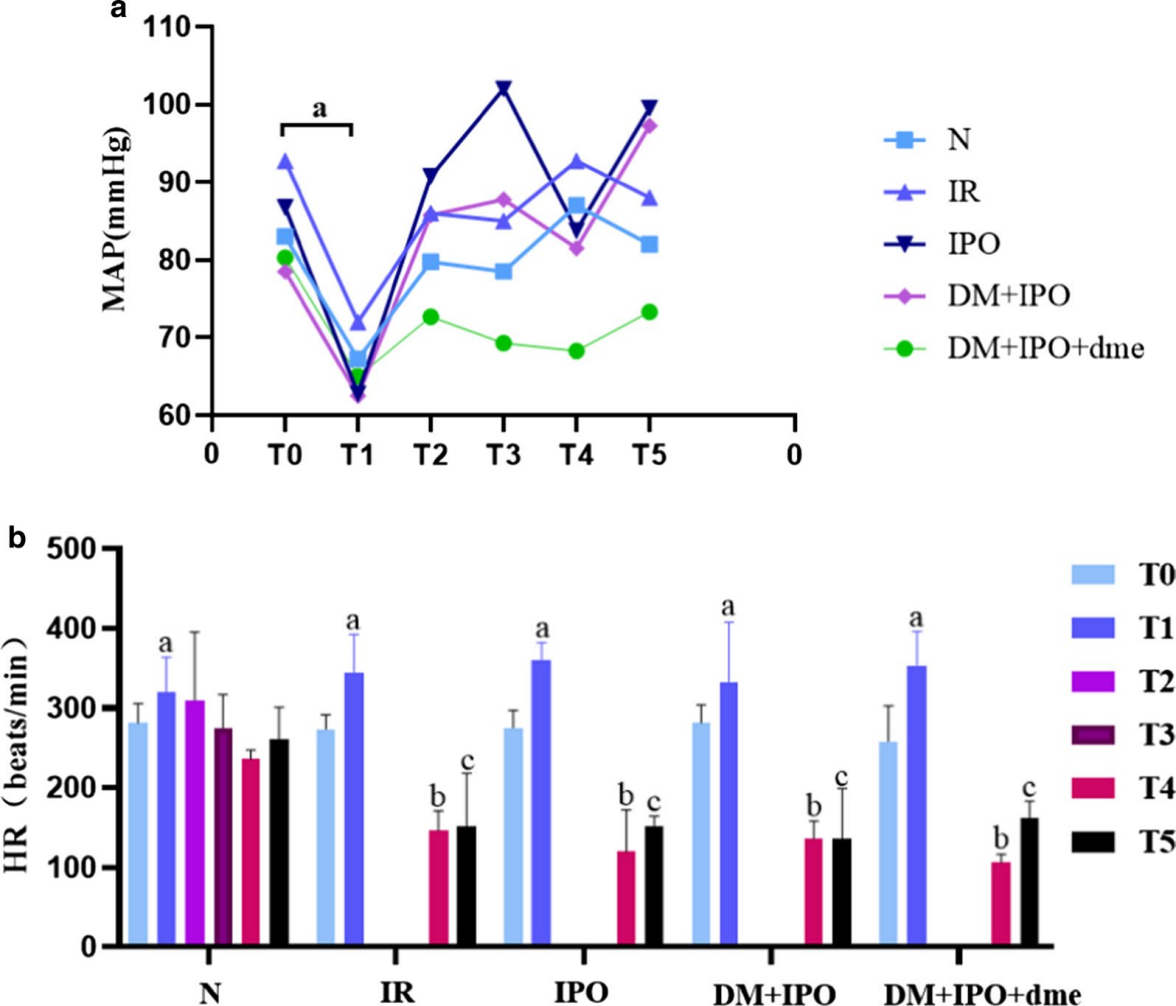
Changes in the heart function of rats from each group. **a** Changes of the MAP in rats in each group. **b** Changes in the HR in-vivo rats in each group. T0, 5 min before CPB; T1, at the beginning of CPB; T2, at cardiac arrest; T3, at the end of cardiac arrest; T4, after 5 min of reperfusion; T5, at the end of reperfusion. ^a^P < 0.05 versus T0; ^b^P < 0.05 versus. T4 in the N group; ^c^P < 0.05 versus T5 in the N group. *MAP* mean arterial pressure, *DM* diabetes mellitus, *HR* heart rate, *N* normal, *IR* ischemia–reperfusion, *IPO* ischemia post-conditioning, *DM* + *IPO* DM ischemia post-conditioning; *DM* + *IPO* + *dme* DM ischemia post-conditioning with inhibitor

As compared with T0, the MAP in each group was significantly reduced at T1 (P < 0.05; Fig. 3A).

#### Changes in the HR of rats in each group

As compared with T0, the HR in each group was significantly increased at T1 (P < 0.05). The HR in all but the N group was significantly lower at T4 and T5, when compared to T0 (P < 0.05). At T4 and T5, the HR in the IR, IPO, DM + IPO and DM + IPO + dme groups was significantly lower than that in the N group (P < 0.05; Fig. 3B).

### Arterial blood gas analysis results

**Fig. 4:**
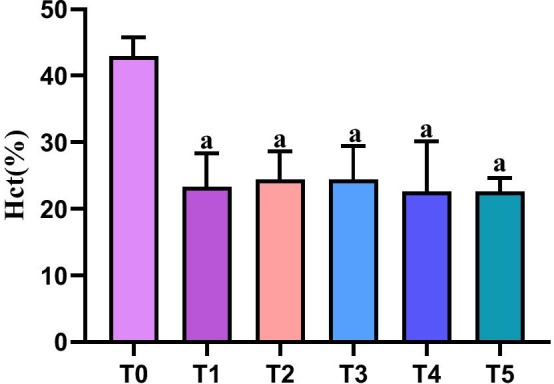
Changes in the HCT of rats in each group. T0, 5 min before CPB; T1, at the beginning of CPB; T2, at cardiac arrest; T3, at the end of cardiac arrest; T4, after 5 min of reperfusion; T5, at the end of reperfusion. ^a^P < 0.05 versus T0. *HCT* haematocrit, *CPB* cardiopulmonary bypass

As compared with T0, the hematocrit (HCT) at each time point of T1-T5 was significantly reduced (P < 0.05). The remaining indicators were within the normal range (Table 1; Fig. 4).

**Table 1:** Changes in blood gas analysis indexes in normal rats

Index	T0	T1	T2	T3	T4	T5
RT (°C)	37 ± 0	37 ± 0	30 ± 0	30 ± 0	37 ± 0	37 ± 0
pH	7.39 ± 0.03	7.36 ± 0.01	7.43 ± 0.07	7.39 ± 0.04	7.4 ± 0.05	7.43 ± 0.03
PaCO_2_	43.3 ± 3.44	42.18 ± 1.75	41.3 ± 2.84	41.9 ± 2.84	42.08 ± 2.22	41.8 ± 1.91
PaO_2_	297.33 ± 35.21	240 ± 68.35	237.6 ± 81.05	221.8 ± 101.5	219.6 ± 26.6	250 ± 42.31
HCT (%)	42.88 ± 2.91*	23.32 ± 4.99*	24.36 ± 4.22*	24.38 ± 5.09*	22.56 ± 7.56*	22.6 ± 2.06*
K^+^	3.82 ± 0.26	3.9 ± 0.29	3.9 ± 0.31	3.99 ± 0.27	3.96 ± 0.28	3.97 ± 0.4
BE	1.92 ± 0.36	1.67 ± 0.51	2.16 ± 0.5	1.3 ± 0.67	1.36 ± 0.36	0.78 ± 1.59

T0, 5 min before CPB; T1, at the beginning of CPB; T2, at cardiac arrest; T3, at the end of cardiac arrest; T4, after 5 min of reperfusion; T5, at the end of reperfusion

*P < 0.05 versus T0

### Comparison of MI area

**Fig. 5:**
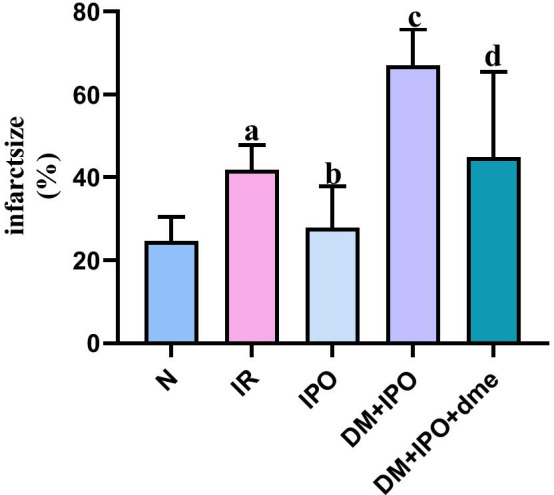
Comparison of isolated heart infarction area of rats in each group at the end of reperfusion. ^a^P < 0.05 vs. the N group; ^b^P < 0.05 versus the IR group; ^c^P < 0.05 versus the IPO group; ^d^P < 0.05 versus the DM + IPO group. *N* normal, *IR* ischemia–reperfusion, *IPO* ischemia post-conditioning, *DM* + *IPO* DM ischemia post-conditioning, *DM* + *IPO* + *dme* DM ischemia post-conditioning with inhibitor

At T5, the MI area in the IR group was significantly increased (P < 0.05), as compared with the N group. As compared with the IR group, the MI area in the IPO group decreased (P < 0.05). As compared with the IPO group, the MI area in the DM + IPO group was higher (P < 0.05). As compared with the DM + IPO group, the MI area in the DM + IPO + dme group decreased (P < 0.05; Fig. 5).

### Changes in serum CK-MB and CTnI levels

#### CK-MB

**Fig. 6:**
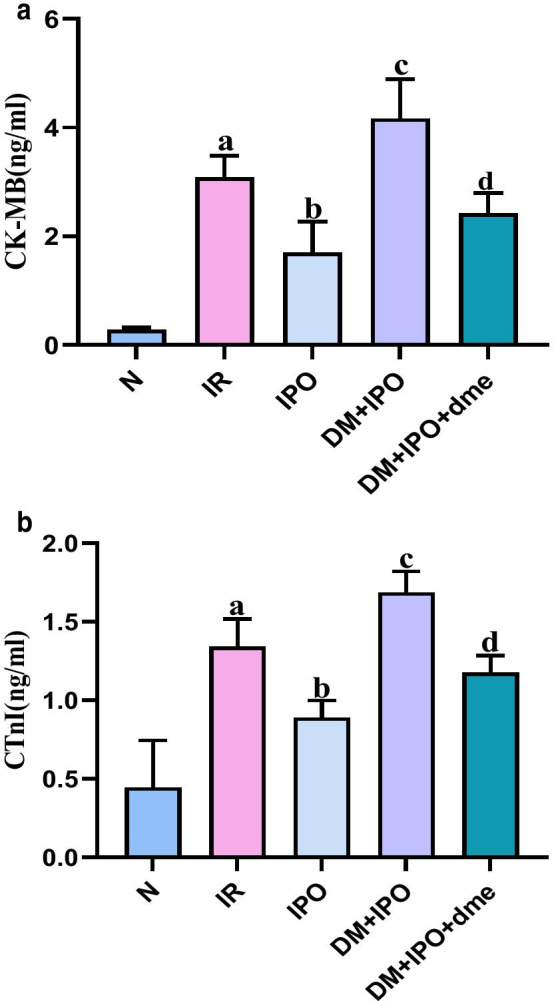
Changes in serum CK-MB and CTnI levels. **a** Changes in the serum CKMB content. **b** Changes in the serum CTnI content vs. the N group. ^a^P < 0.05 versus the N group; ^b^P < 0.05 versus the IR group; ^c^P < 0.05 versus the IPO group; ^d^P < 0.05 versus the DM + IPO group. *CTnl* cardiac troponin, *CK-MB* creatine kinase isoenzyme, *N* normal, *IR* ischemia–reperfusion, *IPO* ischemia post-conditioning, *DM* + *IPO* DM ischemia post-conditioning, *DM* + *IPO* + *dme* DM ischemia post-conditioning with inhibitor

At T5, the CK-MB content in the IR group was significantly higher than that of the N group (P < 0.05). The CK-MB content in the IPO group was lower than that in the IR group (P < 0.05). The DM + IPO group was higher than that of the IPO group (P < 0.05). The DM + IPO + dme group was lower than that in the DM + IPO group (P < 0.05; Fig. 6A).

#### CTnI

At T5, the IR group was significantly higher than the N group (P < 0.05), and the IPO group was lower than the IR group (P < 0.05). The DM + IPO group was higher than the IPO group (P < 0.05). The DM + IPO + dme group was lower than the DM + IPO group (P < 0.05; Fig. 6B).

### Changes in the relative expression of SDHA mRNA in rat myocardial tissue detected by RT-qPCR

**Fig. 7:**
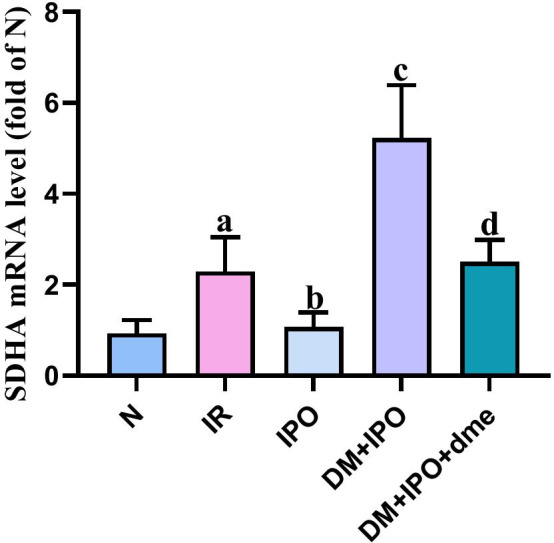
Ratio of target heart SDHA to internal reference gene GAPDH mRNA in each group. ^a^P < 0.05 versus the N group; ^b^P < 0.05 versus the IR group; ^c^P < 0.05 versus the IPO group; ^d^P < 0.05 versus the DM + IPO group. *DM* diabetes mellitus, *SDHA* dehydrogenase-flavin protein, *N* normal, *IR* ischemia–reperfusion, *IPO* ischemia post-conditioning, *DM* + *IPO* DM ischemia post-conditioning, *DM* + *IPO* + *dme* DM ischemia post-conditioning with inhibitor

At T5, as compared with the N group, the relative mRNA expression of SDHA in the IR group was significantly increased (P < 0.05). As compared with the IR group, the relative mRNA expression of SDHA in the IPO group decreased (P < 0.05). As compared with the IPO group, the relative mRNA expression of SDHA in the DM + IPO group increased (P < 0.05). As compared with the DM + IPO group, the relative mRNA expression of SDHA in the DM + IPO + dme group decreased (P < 0.05; Fig. 7).

### The relative protein expression of SDHA was detected by western blotting

**Fig. 8:**
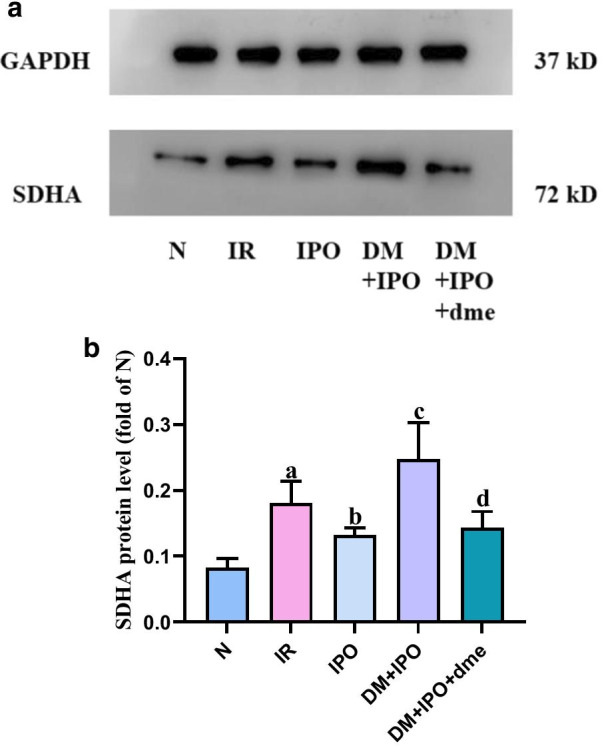
Western blotting was used to detect the protein expression of SDHA in the myocardium of each group. **a** Expression of SDHA and GAPDH proteins in the heart of rats in each group. **b** Ratio of SDHA to GAPDH of target protein of rat heart in each group. ^a^P < 0.05 versus the N group; ^b^P < 0.05 versus the IR group; ^c^P < 0.05 versus the IPO group; ^d^P < 0.05 versus the DM + IPO group. *DM* diabetes mellitus, *SDHA* dehydrogenase-flavin protein, *N* normal, *IR* ischemia–reperfusion, *IPO* ischemia post-conditioning, *DM* + *IPO* DM ischemia post-conditioning, *DM* + *IPO* + *dme* DM ischemia post-conditioning with inhibitor. Full-length gels are presented in Supplementary file

At T5, the relative protein expression of SDHA in the IR group was significantly higher than that in the N group (P < 0.05). As compared with the IR group, the relative protein expression of SDHA in the IPO group decreased (P < 0.05). As compared with the IPO group, the relative protein expression of SDHA in the DM + IPO group increased (P < 0.05). As compared with the DM + IPO group, the relative protein expression of SDHA in the DM + IPO + dme group decreased (P < 0.05; Fig. 8).

## Discussion

In the present study, it was found that the blood glucose level of DM rats peaked following oral administration and injection of a 20% GS solution, with the blood glucose level subsequently remaining high. At the same time, DM rats also showed symptoms of “polydipsia, polyphagia, polyuria and weight loss” and experienced increased orbital secretions and ulcerations of the tail skin. These results showed that the DM rat model established in experiment was effective and successfully.

The arterial blood gas test is a common diagnostic method in clinically [21]. In this experiment, the HCT values of rats in the N group decreased at T1-T5, as compared with T0. The reason was that, following the start of CPB, the priming fluid diluted the blood, which caused a decrease in HCT; moreover, in the experiment allogeneic rat blood was not added to the pre-filled solution, which avoided the loss of body fluid due to blood type discrepancies. The other blood gas indicators, such as pH, PaCO_2_, PaO_2_ and K^+^, were all within the normal ranges. Studies have reported that dme at a dose of 4 mg/kg·min^−1^ can reduce the MI area in IR rats and exert myocardial protection. Therefore, the present study also adopted the same dose [22].

IPGTT and OGTT experiments confirmed the reliability of the DM rat model. Arterial blood gas analysis confirmed that the rat’s CPB model established in this experiment was successful. They laid the foundation for the further steps.

### The relationship between IPO and MIRI in the normal heart

The pathogenesis of MIRI is multi-factorial, such as calcium overload, oxidative stress, endoplasmic reticulum stress, mitochondrial dysfunction were all interrelated [23–25]. In 2003, Zhao et al. proposed that IPO is also an effective method to protect the ischemic myocardium from MIRI [26], the mechanism, which IPO exerts protection may be due to the fact that IPO itself can delay sympathetic nerve activation during ischemia and reduce the opening of mitochondrial KATP-sensitive potassium channels [27], as well as increase the phosphorylation level of endothelial nitric oxide synthase to protect the myocardium from damage [28].

The present study found that, following the occurrence of MIRI, the content of CK-MB and CTnI increased significantly, and the MI area increased significantly, indicating that IPO can cause an increase in the MI area and serum MI markers, and cause myocardial damage. These specific biomarkers play a key role in the early diagnosis of acute MI and good indicators of patients recovery [29]. This was consistent with the results of several studies [30–32].

SDHA plays an important role in the tricarboxylic acid cycle (TCA) and aerobic respiratory chain systems. This is because, first, SDHA can catalyze the oxidation process of succinic acid to fumaric acid in the TCA, and couple with coenzyme Q [33]. Secondly, SDHA is the only shared enzyme in the respiratory chain [34]. This study found that, in the IR group, the SDHA mRNA and protein relative expression levels were increased, as compared with the N group. The reason may be because IR can cause the respiratory capacity of mitochondria to be disappeared and a large amount of ROS to be produced. At this time, succinic acid and fumaric acid accumulate in large, the hypoxia signaling pathway is opened, and vascular inflammation appears [35]. The above processes together lead to the death of cardiomyocytes, increase the relative expression of SDHA mRNA and protein, and trigger MIRI finally.

However, when comparing the IR with the IPO group, it was found that the relative expression of SDHA in the latter group was reduced. At this time, the serum CK-MB and CTnI levels decreased, and the MI area decreased. The reason may be because, first, IPO can reduce vascular inflammation and endothelial dysfunction, and inhibit the excessive activation of the renin–angiotensin–aldosterone system. Secondly, IPO can scavenge the generation of free radicals and reduce the oxidative stress caused by IR, thereby reducing the damage of the myocardial inner membrane and leakage of myocardial enzymes [36]. In conclusion, we believe that IPO can inhibit the relative mRNA and protein expression of SDHA and reduce MIRI.

### Relationship between IPO and MIRI in the DM myocardium

Epidemiology has shown that DM can accelerate the incidence and mortality of heart disease [37]. Several studies have mentioned that hyperglycemia affects the sensitivity of the myocardium to MIRI [38–40]. In the present study, it was found that, as compared with the DM group, the DM + IPO group had a higher CK-MB and CTnI content, a larger MI area and more serious myocardial damage. The myocardial protection of IPO was almost ineffective. This is because, When the plasma glucose concentration increases, O-linked β-N-acetylglucosamine (O-GlcNAc) is converted into the monosaccharide donor UDP-N-acetylglucosamine (UDP-GlcNAc) following glycosylation, which participates in the pathological changes of the DM myocardium. The reduced level of O-GlcNAc glycosylation also affects the protective effect of IPO [41, 42]. In addition, the hyperglycemia state continuously activates glycogen synthase kinase-3, which can indirectly activate phosphatidylinositol 3-kinase-Akt, inhibit its phosphorylation, increase the MI area and serum CTnI level in DM rats and aggravate MIRI [43].

The present study found that the relative expression of SDHA mRNA and protein was significantly higher in the DM + IPO than in the IPO group, and the degree of myocardial damage in the DM + IPO group was significantly higher than that in the IPO group. A study has shown that hyperglycemia leads to the complete loss of mitochondrial self-storage breathing capacity [15]. Moreover, the increase in the expression of SDHA has been shown to lead to a large accumulation of succinic and fumaric acid, the opening of the hypoxia signal pathway and the appearance of vasculitis [13]. The above process not only blocks the myocardial protective effect of IPO, but also further increases the sensitivity of the DM myocardium to IR and aggravates tissue damage. This is why the protective effect of IPO in the DM myocardium is weakened or disappears.

### Role of inhibitor dme in the DM myocardium

As a competitive inhibitor of SDH—dme, which can inhibit SDHA expression and reduce brain damage in rats. The underlying mechanisms include inhibiting mitochondrial membrane potential in the cytoplasm from overpolarization, stabilizing the structure of HIF-1α, limit reverse electron transport [16], and ultimately reduce ROS generation [44]. Moreover, dme exerts a cardioprotective effect in DM rats [45]. Unfortunately, this study only conducted experiments on the association between SDHA and dme in isolated rat myocardial tissue, and did not discuss in detail whether dme can inhibit the expression of SDHA and exert myocardial protection under the conditions of rat CPB.

In this experiment, using a rat CPB model, it was found that the expression of SDHA in the DM + IPO + dme group was significantly lower than that in the DM + IPO group. Not only that, the infart area of the DM + IPO + dme group was smaller, and the serum CK-MB and CTnI levels were lower than those in the DM + IPO group. Therefore, the experiment showed that, although the effect of IPO in reducing MIRI in DM rats disappears, dme can inhibit the expression of SDHA, reduce myocardial inflammation with enzyme, and restore the protective effect of IPO against MIRI in DM rats. The results of the present study fully explained that the disappearance of IPO in reducing MIRI in the DM myocardium is associated with SDHA, and inhibiting the expression of SDHA gene and protein is beneficial for IPO to reduce myocardial MIRI in DM.

### The effect of multiple cardiovascular drugs on MIRI

Hyperglycemia can affect the myocardial protection of IPO. In fact, patients with cardiovascular diseases also receive the treatments with aspirin, β-blockers, ACE inhibitors statins and other drugs at the same time. The above drugs may affect the effect of IPO in DM patients, even non-DM patients. Whether to use antiplatelet therapy for patients with type 2 diabetes, this is still a debated issue, because more and more patients are resistant to aspirin [46, 47], which may be due to the imbalance between pro-anti-inflammatory and anti-inflammatory cytokines caused by the changes of P450 genotype in the cytochrome. They leads to impaired of platelet’s aggregation ultimately [48]. There were confirmed by some studies that, compared with non-DM patients, DM people receive less benefit from aspirin [49]. However, some studies have pointed out, the use of aspirin can directly protect the myocardium from IRI [50, 51], it is not to inhibit platelet aggregation, but to trigger the phosphorylation of sphingosine, and form the protective sphingosine-1-phosphate (SP1) finally [52]. Therefore, whether to use of antiplatelet drugs? Is it beneficial or harmful to DM patients? They are worthy of further discussion.

Β-blockers have shown beneficial effects on cardiac dysfunction by scavenging free radicals or acting as antioxidants. The study by Erkan Tuncay et al. pointed out that, the degree of oxidative stress of cardiomyocytes is related to the free Ca^2+^ level in mitochondria. β-blockers such as Timolol may prevent the Ca^2+^ homeostasis by adjusting the level of Zn^2+^, maintaining the redox state and play a protective effect on DM myocardium [53]. ACE inhibitors such as Perindopril can increase the expression of many signaling molecules [54], including stromal cell-derived factor-1α (SDF-1α) and vascular endothelial growth factor (VEGF)[55]. These molecules are released from the ischemic myocardium into blood vessels, and act on the bone marrow to promote the release of endothelial progenitor cells (EPCs) and reduce MIRI finally. Other Secondary prevention drugs for heart disease, such as statins, by activating the PI3/Akt pathway, inducing the differentiation of EPCs, increasing their numbers, and reducing myocardial damage in DM patients [56].

## Conclusions

IPO can inhibit the expression of SDHA, reduce MIRI and exert a cardioprotective effect in the normal rats. However, the protective effect of IPO disappears in the diabetic rats. The inhibitor dme combined with IPO can increase the expression of SDHA and restore the protective effect of IPO in DM myocardia.

## Supplementary Information

**Additional file 1:** The full-length gels of SDHA protein is shown in the supplementary file.

## Data Availability

The data and/or analyzed during the study are available from the corresponding author upon reasonable request..

## Abbreviations

DM: Diabetes mellitus
SDHA: Succinate dehydrogenase-flavin protein
IPO: Ischemic post-processing
Dme: Dimethyl malonate
CPB: Cardiopulmonary bypass
SD rats: Sprague Dawley rats
IPGTT: Intraperitoneal glucose tolerance test
OGTT: Oral glucose tolerance test
CK-MB: Creatine kinase isoenzyme
CTnI: Cardiac troponin
MIRI: Myocardial ischemia–reperfusion injury
MAP: Mean arterial pressure
HR: Heart rate
