# Supplementary material for: The disappearance of IPO in myocardium of diabetes mellitus rats is associated with the increase of succinate dehydrogenase-flavin protein
Source: BMC Cardiovasc Disord. 2021 Mar 17;21:142. doi: 10.1186/s12872-021-01949-z (PMC7968298; doi:10.1186/s12872-021-01949-z)

The disappearance of IPO in myocardium of diabetes mellitus rats is associated with the increase of succinate dehydrogenase-flavin protein

MENGYUAN DENG[1*], WEI CHEN[1*], HAIYING WANG[1], YAN WANG[1], WENJING ZHOU[2] and TIAN YU[3]

[1]Department of Anesthesiology, Affiliated Hospital of Zunyi Medical University, Zunyi, Guizhou, 563003; [2]Anesthesia laboratory, Affiliated Hospital of Zunyi Medical University, Zunyi; [3]Zunyi Medical University, Zunyi, P.R. China

[*]Contributed equally

Correspondence to: Professor Haiying Wang, Department of Anesthesiology, Affiliated Hospital of Zunyi Medical University, 563003; 149 Dalian Road, Huichuan District, Zunyi, Guizhou 563003, P.R China.

E-mail: wanghaiting-8901@163.com

Figure 1

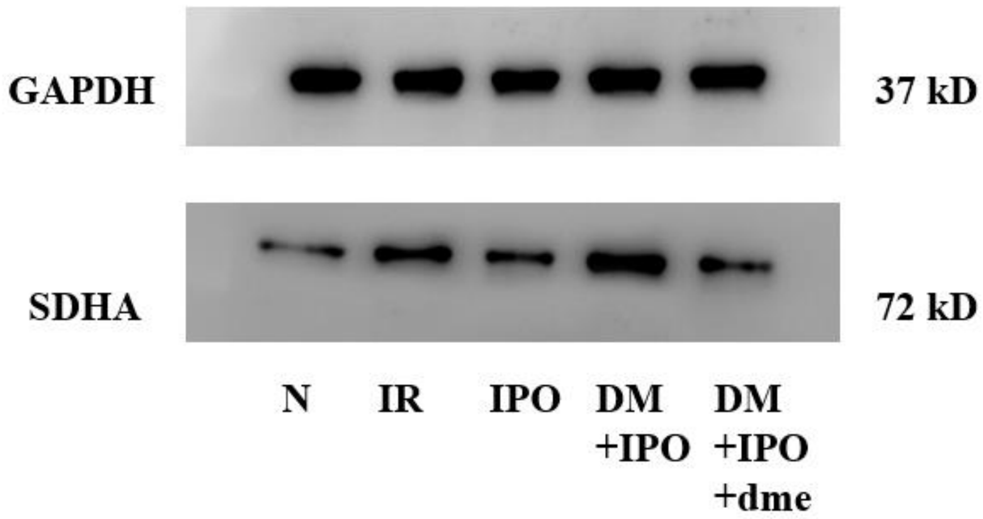

The orinal image without crop and correction as follows.

GAPDH:

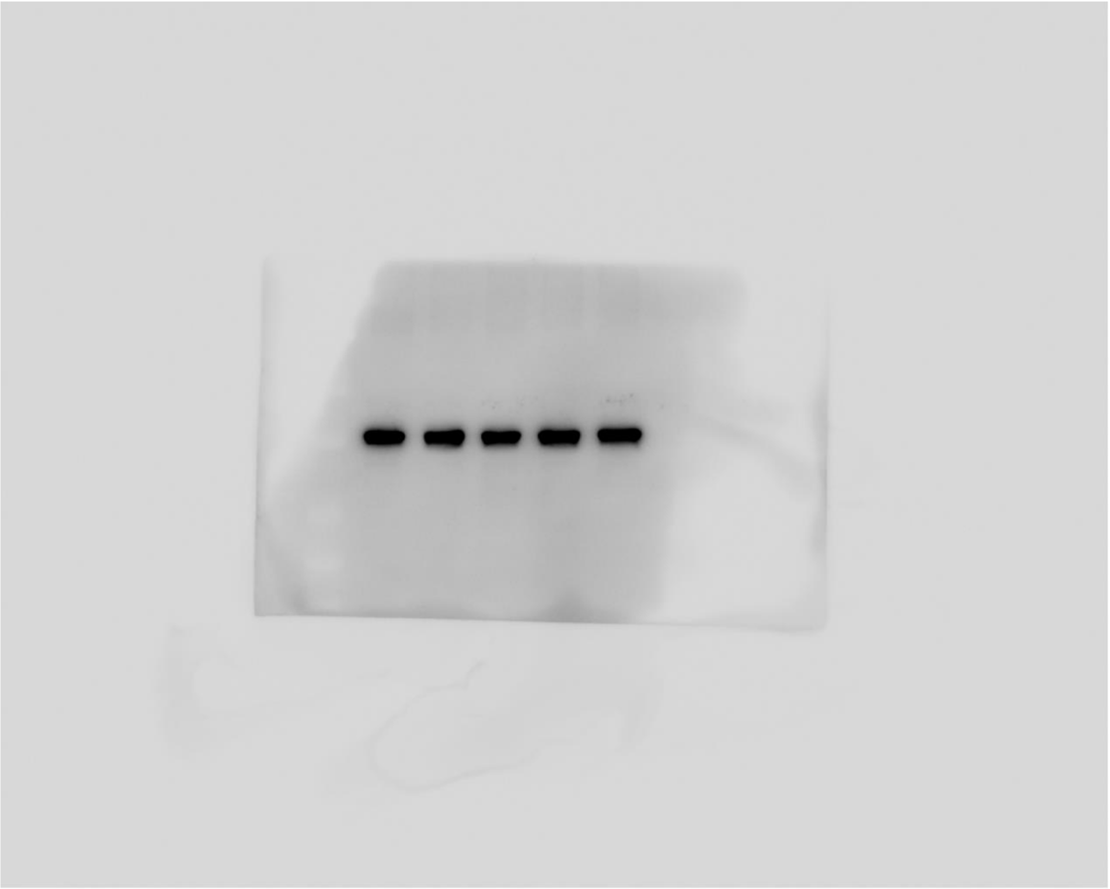

SDHA

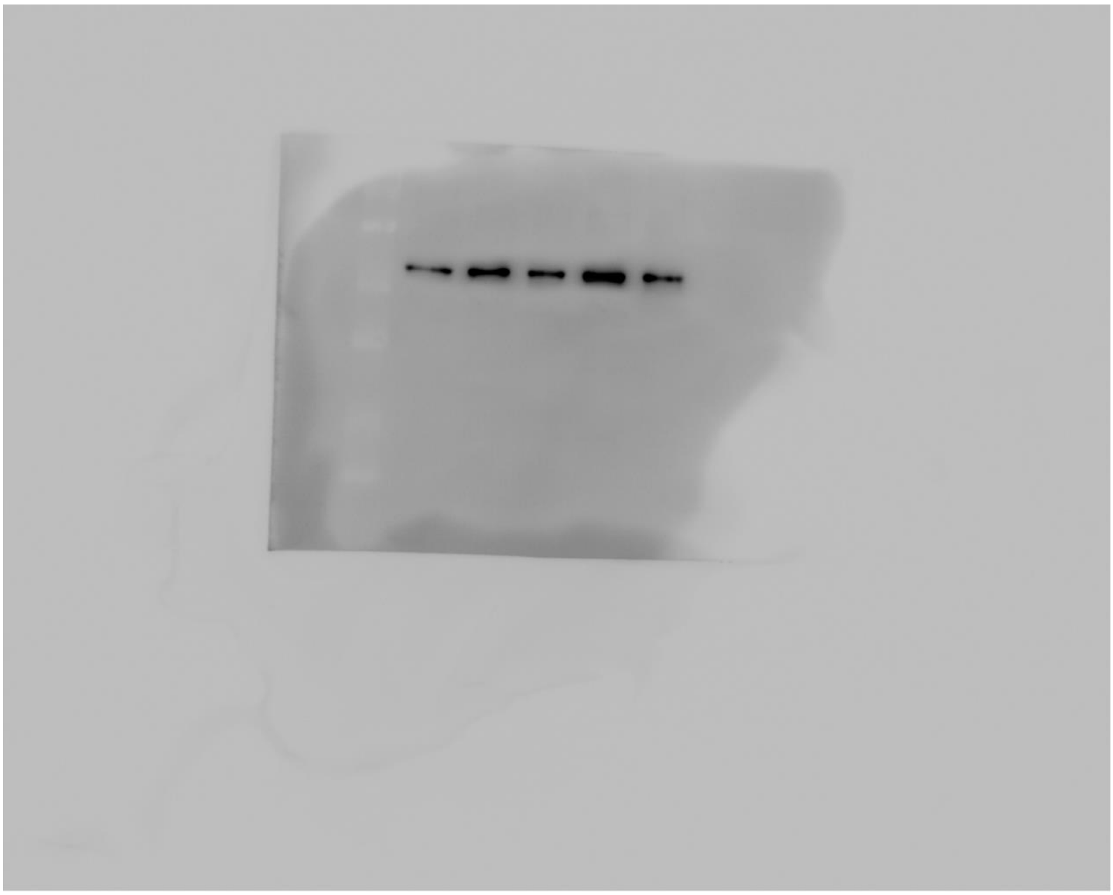

Supplement: Supplementary file 1 — Additional file 1: The full-length gels of SDHA protein is shown in the supplementary file. [file 12872_2021_1949_MOESM1_ESM.pdf]
